# Consistency of patient-reported outcomes after cholecystectomy and their implications on current surgical practice: a prospective multicenter cohort study

**DOI:** 10.1007/s00464-016-4959-x

**Published:** 2016-05-18

**Authors:** Sarah Wennmacker, Mark Lamberts, Jos Gerritsen, Jan Anne Roukema, Gert Westert, Joost Drenth, Cornelis van Laarhoven

**Affiliations:** 1 Department of Surgery, Radboud University Medical Center, PO Box 9101, 6500 HB Nijmegen, The Netherlands; 2 Department of Gastroenterology and Hepatology, Radboud University Medical Center, Nijmegen, The Netherlands; 3 Department of Surgery, Medisch Spectrum Twente, Enschede, The Netherlands; 4 Department of Surgery, St. Elisabeth Hospital, Tilburg, The Netherlands; 5 Scientific Institute for Quality of Healthcare (IQ Healthcare), Radboud University Medical Center, Nijmegen, The Netherlands

**Keywords:** Cholecystolithiasis, Gallstones, Cholecystectomy, Patient-reported outcomes, Selection criteria

## Abstract

**Background:**

Persistent postoperative pain (up to 41 %) and significant practice variation necessitate better patient selection for cholecystectomy. Patient-reported outcome measures (PROMs) are nowadays known to serve as a tool for better patient selection, although variability within these subjective outcomes may be a point for debate. This study determines associations of both the preoperative pain and patient characteristics with PROMs at 24 weeks after cholecystectomy. In order to evaluate variability of PROMs, we also determined consistency of these outcomes in time.

**Methods:**

This prospective multicenter cohort study included adult patients diagnosed with uncomplicated symptomatic cholecystolithiasis. Twenty-four weeks after surgery, a questionnaire study was carried out, containing Gastrointestinal Quality of Life Index (GIQLI) and Patients’ Experience of Surgery Questionnaire. Results were compared to preoperative data and results 12 weeks post-cholecystectomy. Logistic regression analyses were performed to determine associations. Additional post hoc analysis on associations between preoperative selection criteria and PROMs was done.

**Results:**

A total of 360 patients (85 %) responded. Postoperative absence of pain was reported by 59.2 %. Associated characteristics were symptoms ≤1 year prior to surgery [OR 1.85 (95 % CI 1.11–3.09)] and high baseline GIQLI score [OR 1.04 (95 % CI 1.02–1.05)]. General improvement in abdominal symptoms and positive result of surgery were found in 90 %; no preoperative variables were significantly associated. PROMs showed consistency at 12 and 24 weeks postoperatively. Post hoc analysis showed no significant associations.

**Conclusion:**

PROM-based preoperative selection criteria need to be considered to select those patients who achieve freedom of pain after surgical treatment of uncomplicated symptomatic cholecystolithiasis. Other patients might consider cholecystectomy as successful, but are less likely to be free of pain. Usefulness of PROMs is underscored as they proved to be consistent in time in evaluating surgical outcome.

Cholecystectomy is the current gold standard for symptom relief in uncomplicated symptomatic cholecystolithiasis [1–3]. However, cholecystectomy is not always required because in up to 31 % of patients, watchful waiting results in adequate symptom control [4]. The decision to perform cholecystectomy is based on the presence of a biliary colic defined by the Rome III criteria [5, 6]. Most patients do not give a history of a biliary colic but report nonspecific abdominal symptoms [7]. Vice versa, up to 41 % of patients still report pain post-cholecystectomy, suggesting inefficient use of cholecystectomy [8, 9]. So far it has been difficult to address the importance of this issue in the surgical world, as well as the realization that a 60 % benefit rate from a surgical treatment is insufficient in a cash-strapped healthcare environment. The ineffective matching of symptoms and procedure leads to practice variation. This arises when there is uncertainty about the best option for effective treatment or when different criteria are applied in the decision to perform surgery [10–12]. Practice variation is ubiquitous for cholecystectomy [13, 14].

The combination of large practice variation and significant persistence of postoperative pain emphasizes the need for more evidence on proper patient selection for cholecystectomy. Persistence of pain after surgery is possibly due to wrong symptom attribution to gallstones or newly arisen symptoms after cholecystectomy [9]. Only when we acknowledge the shortcomings of the clinical thinking process that leads up to the indication of a cholecystectomy, we can move to a better use of cholecystectomy. Associations between preoperative characteristics and patient-reported outcomes (PROMs) might help to identify the proportion of patients who truly will benefit from surgery. Preoperative pain and patient characteristics correlate with patient-reported pain relief after cholecystectomy [15–19]. However, variation of PROMs has been reported in the literature, and timing and frequency after surgery remain controversial [20, 21]. Therefore, associations based on PROMs need to be replicated in independent studies with a time-dependent granularity to judge development over time.

We aimed to determine the association of preoperative pain and patient characteristics with patient-reported postoperative absence of abdominal pain, improvement in abdominal symptoms in general and success of surgery at 24 weeks after elective cholecystectomy in uncomplicated symptomatic cholecystolithiasis patients. We also aimed to assess the consistency of PROMs after cholecystectomy at 12 and 24 weeks after surgery.

## Methods

### Study site and subject selection

This prospective cohort study was conducted at the Radboudumc Nijmegen, St. Elisabeth Hospital Tilburg and Medisch Spectrum Twente Enschede, The Netherlands. Details of the study design were reported previously [19]. Briefly, included patients visited the outpatient clinic of the surgery departments between June 2012 and June 2014. Patients had to be 18 years of age or older, classified as American Association of Anesthesiologists (ASA) I or II and scheduled for elective laparoscopic cholecystectomy based on a diagnosis of uncomplicated symptomatic cholecystolithiasis. Patients underwent a structured anamnesis at the outdoor clinic and were thereafter asked to complete questionnaires preoperatively [Gastrointestinal Quality of Life Index (GIQLI) and McGill Pain Questionnaire (MPQ)] and 12 weeks postoperatively [GIQLI and Patients’ Experience of Surgery Questionnaire (PESQ)].

We report the second part of the prospective cohort study, in which all patients that had previously returned the preoperative questionnaire were sent a second postoperative questionnaire 24 weeks after surgery. Patients who did not return this questionnaire were contacted. Those who subsequently completed the questionnaire were classified as “late responder.” All non-responders were excluded from analysis.

The questionnaire consisted of the GIQLI [22, 23] and PESQ [24], both translated and validated in Dutch. The GIQLI contains questions on gastrointestinal symptoms for the upper and lower digestive tract and general, physical, emotional and social functioning in the last 2 weeks. Each question can be scored from 0 to 4 (0 being the worst and 4 the best appraisal). An overall score of 0–144 can be calculated; the lower the score, the lower the health status. The PESQ contains questions on complications, postoperative abdominal symptoms and patient-reported results of surgery. The latter two questions consisted of five response categories.

The study was approved by the medical ethics committee of all hospitals and was performed in accordance with the Declaration of Helsinki and with the recommendations in the Strengthening the Reporting of Observational Studies in Epidemiology (STROBE) guidelines for reporting observational studies [25].

### Outcome and variables of interest

The primary outcome was defined as the absence of abdominal pain 24 weeks after cholecystectomy as reported on the postoperative GIQLI. We dichotomized results in the absence or presence of abdominal pain. Secondary outcomes were patient-reported general improvement in abdominal symptoms and result of surgery 24 weeks after cholecystectomy. We dichotomized results in improvement (much better, slightly better) or absence of improvement (the same, slightly worse or much worse) in abdominal symptoms after cholecystectomy and good (excellent, very good and good) and bad (bad and very bad) results of surgery. We estimated the association of preoperative pain characteristics, patient characteristics and health status with both primary and secondary outcomes. Finally, all outcomes 24 weeks after cholecystectomy and associated variables were compared to the previous 12-week follow-up results. Similarities and differences in the results were described.

Based on previous publications [16–18], we included independent variables gender, age at time of surgery, study center, preoperative absence of abdominal pain (last 2 weeks, as reported on GIQLI), ASA classification, duration of symptoms ≤1 year, baseline GIQLI score, pain-induced awakening at night, severity of pain and pain occurring in episodes.

### Power analysis

The appropriate sample size for the study was calculated based on findings of a systematic review [9]. Persistence of postoperative pain was estimated at 33 % with an error rate of 5 and 95 % confidence interval (CI). This resulted in total sample size of 340 patients, with an estimated 112 patients in the smallest response group (persistence of postoperative pain). This number is sufficient to analyze 11 variables in multivariate analysis. Anticipating on a response rate of 60 %, at least 476 needed to be invited for participation [19]. In total, 552 patients were included, of whom 423 returned their preoperative questionnaire. The latter 423 patients were contacted for part of the study for follow-up at 24 weeks after surgery. As these patients had previously returned a questionnaire, response rate was estimated at 80 %, again resulting in sufficient observations to analyze 11 variables in multivariate analysis.

### Statistical analysis

To examine significant differences in patient characteristics between responders (including late responders) and non-responders, Chi-square test or Fisher’s exact test for categorical data and Student’s t test for continuous data were used.

To identify which variables were associated with primary and secondary outcomes, logistic regression analysis was applied. Variables with a *p* value <0.1 in univariable analyses were included in multivariable logistic regression analysis. Backward elimination was used as variable selection method retaining age at time of surgery, gender, study center and preoperative absence of abdominal pain as covariables. The results were reported as adjusted odd ratios (OR) and 95 % CIs. To determine whether the PROMs significantly differed between 12 and 24 weeks after cholecystectomy, McNemar’s test was used. A *p* value <0.05 was considered statistically significant. All missing values were considered to be at random and were excluded from analyses. All statistics were done with SPSS statistics 20.0 (IBM).

### Post hoc analysis

Based on the results of the regression analyses, we performed a post hoc analysis on the association between the preoperative presence of biliary colic and postoperative PROMs. Since biliary colic is in daily practice defined in different ways, we explored these associations with four commonly used definitions of biliary colic; episodic pain in epigastrium or right upper abdominal quadrant with a duration of at least 30 min without additional features (1), with pain radiating to the back (2), with urge to move (3) and with both pain radiating to the back and urge to move (4). Information on the presence of biliary colic was abstracted from the structured anamnesis. Associations between the presence of biliary colic preoperative and postoperative PROMs were analyzed using univariate and multivariate logistic regression analysis.

## Results

### Baseline characteristics

We send follow-up questionnaire 24 weeks after surgery to 423 patients. Questionnaires were returned by 360 (85 %) patients: 314 (87.2 %) were returned immediately after 6 months and another 46 (12.8 %) late responders between 8 and 25 months postoperatively. Baseline characteristics of the responders and non-responders are shown in Table 1. The proportion of female responders was 77.5 % (*n* = 279). Mean age at surgery was 49.8 years (SD 14.2 years). None of the baseline characteristics of the responders were different from characteristics of non-responders, except for gender and age. Non-responders were more often female (88.9 % *p* = 0.04) and were younger (43.6 SD 15.6, *p* = 0.002).

**Table 1:** Baseline characteristics responders and non-responders

Characteristics	Responders	Non-responders	*p* value
Gender, M/F (%F)	81/279 (77.5)	7/56 (88.9)	0.04
Age (years), mean (SD)	49.8 (14.2)	43.6 (15.6)	0.002
ASA I/II (%II)	169/191 (53.1)	34/29 (54.0)	0.30
Center general/academic (% academic)	289/71 (17.2)	54/9 (16.7)	0.31

*SD* standard deviation*, ASA* American Society of Anesthesiologists classification

### Associations with the absence of pain, general improvement in abdominal symptoms and result of surgery

**Fig. 1:**
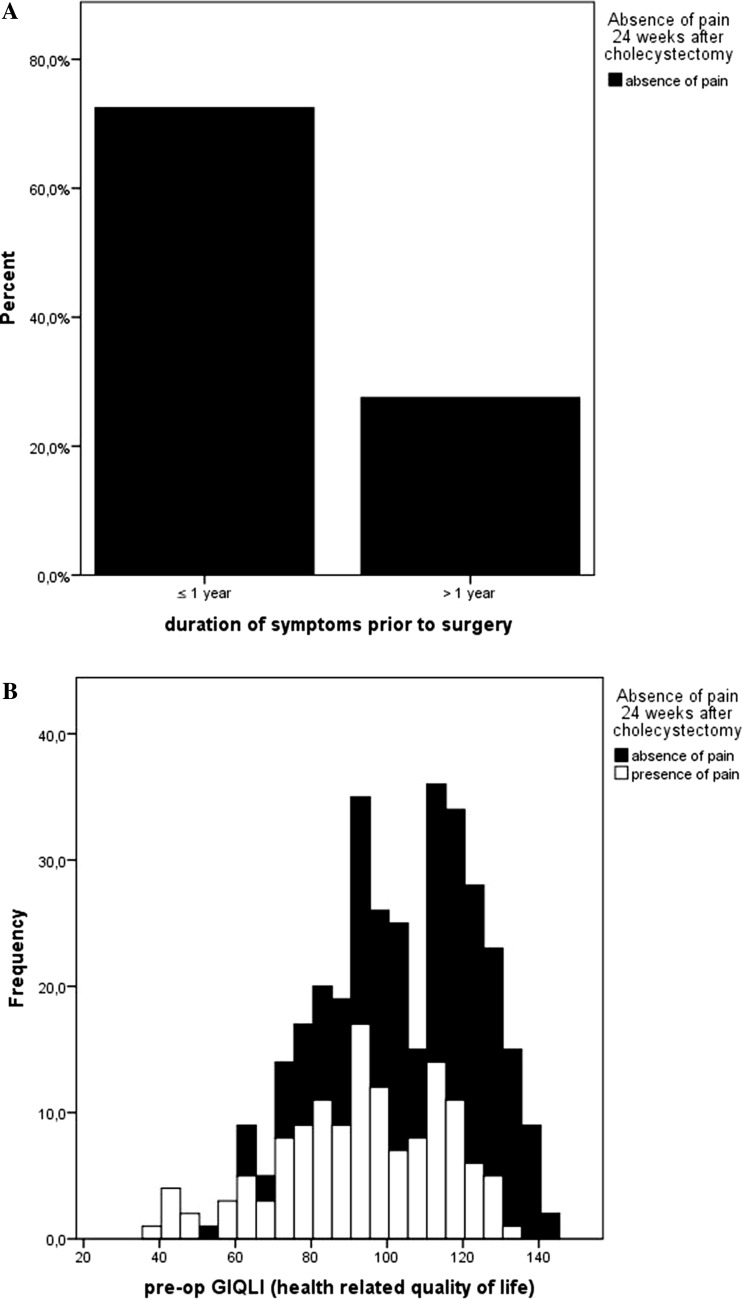
Associations of variables with the absence of abdominal pain 24 weeks after cholecystectomy. **A** Associations between duration of symptoms prior to cholecystectomy and the absence of abdominal pain. OR 1.85, 95 % CI 1.11–3.09, *p* = 0.02. **B** Associations between preoperative GIQLI score and the absence of abdominal pain. OR 1.04, 95 % CI 1.02–1.05, *p* < 0.001

A total of 213 (59.2 %) patients reported postoperative absence of abdominal pain using the GIQLI. Duration of symptoms ≤1 year prior to surgery, awaking at night and baseline GIQLI score were univariably associated (Table 2). After adjustment for age at time of surgery, gender, study center and preoperative absence of abdominal pain in multivariable analysis, duration of symptoms ≤1 year (OR 1.85 95 % CI 1.11–3.09, *p* = 0.02) and baseline GIQLI score (OR 1.04 95 % CI 1.02–1.05, *p* < 0.001) remained significantly associated with the absence of abdominal pain after cholecystectomy (Fig. 1).

**Table 2:** Univariable and multivariable associations of pain and patient characteristics with patient-reported postoperative absence of abdominal pain

Feature	Absence of pain	Presence of pain	Univariable		Multivariable	
			Odds ratio (95 % CI)	*p* value	Odds ratio (95 % CI)	*p* value
Gender [*n* (%)]						
Female	157 (73.7)	121 (82.9)	0.58 (0.34–0.98)	0.04	0.66 (0.36–1.20)	0.17
Male	56 (26.3)	25 (17.1)	1.00 (reference)			
Age (years), mean (SD)	49.7 (13.8)	49.7 (14.7)	1.00 (0.99–1.02)	1.00	1.00 (0.98–1.02)	0.91
Center [*n* (%)]						
Academic	42 (19.7)	29 (11.5)	0.99 (0.58–1.68)	0.99	0.79 (0.43–1.43)	0.43
General	171 (80.3)	117 (78.5)	1.00 (reference)			
Preoperative pain in last 2 weeks [*n* (%)]						
Absence	38 (17.5)	11 (8.2)	2.68 (1.32–5.44)	0.006	1.00 (0.43–2.32)	0.99
Presence	174 (82.5)	135 (91.8)	1.00 (reference)			
ASA [*n* (%)]						
I	107 (50.2)	62 (42.5)	1.00 (reference)			
II	106 (49.8)	84 (57.5)	0.74 (0.48–1.13)	0.16		
Duration of symptoms [*n* (%)]						
≤1 year	150 (72.5)	90 (62.5)	1.57 (1.00–2.48)		1.85 (1.11–3.09)	0.02
>1 year	57 (27.5)	54 (37.5)	1.00 (reference)	0.05		
Awaking at night [*n* (%)]						
Yes	102 (49.5)	98 (67.2)	1.00 (reference)	0.001		
No	104 (50.5)	48 (32.8)	2.08 (1.34–3.23)			
Severity [*n* (%)]						
≤50/100	14 (7.0)	11 (7.9)	1.00 (reference)			
>50/100	186 (93.0)	129 (92.1)	1.13 (0.50–2.58)	0.77		
Baseline GIQLI, mean (SD)	108.2 (19.6)	93.6 (21.3)	1.04 (1.02–1.05)	<0.001	1.04 (1.02–1.05)	<0.001
Type of pain [*n* (%)]						
Episodic	124 (59.9)	77 (47.3)	1.34 (0.87–2.05)	0.18		
Non-episodic	83 (40.1)	69 (52.7)	1.00 (reference)			

*SD* standard deviation*, ASA* American Society of Anesthesiologists classification, *GIQLI* Gastrointestinal Quality of Life Index

Abdominal symptoms in general were reported as “much better” or “slightly better” by 324 (90.0 %) patients after cholecystectomy. No variables were associated with improvement in abdominal symptoms in general. Postoperative absence of abdominal pain itself was both univariably (Table 3) and multivariably (OR 4.90, 95 % CI 2.07–11.60, *p* < 0.001) associated with general improvement in abdominal symptoms.

**Table 3:** Univariable and multivariable associations of pain and patient characteristics with patient-reported general improvement in abdominal symptoms

Feature	Improvement symptoms	No improvement/impairment symptoms	Univariable		Multivariable	
			Odds ratio (95 % CI)	*p* value	Odds ratio (95 % CI)	*p* value
Gender [*n* (%)]						
Female	251 (77.4)	26 (81.3)	0.79 (0.32–2.00)	0.62	0.61 (0.22–1.70)	0.35
Male	73 (26.6)	6 (18.7)	1.00 (reference)			
Age (years), mean (SD)	49.4(14.1)	53.3 (15.0)	0.98 (0.96–1.00)	0.14	0.98 (0.95–1.00)	0.13
Center [*n* (%)]						
Academic	64 (19.8)	6 (18.7)	1.07 (0.42–2.70)	0.89	1.06 (0.40–2.81)	0.91
General	260 (80.2)	26 (81.3)	1.00 (reference)			
Preoperative pain in last 2 weeks [*n* (%)]						
Absence	38 (11.8)	10 (31.2)	0.29 (0.13–0.67)	0.003	0.17 (0.07–0.44)	<0.001
Presence	284 (88.2)	22 (68.8)	1.00 (reference)			
ASA [*n* (%)]						
I	155 (47.8)	12 (37.5)	1.00 (reference)			
II	169 (52.2)	20 (62.5)	0.65 (0.31–1.38)	0.27		
Duration of symptoms [*n* (%)]						
≤1 year	212 (67.3)	25 (78.1)	0.58 (0.24–1.38)	0.22		
>1 year	103 (32.7)	7 (21.9)	1.00 (reference)			
Awaking at night [*n* (%)]						
Yes	181 (57.3)	17 (53.1)	1.00 (reference)			
No	135 (42.7)	15 (46.9)	0.85 (0.41–1.75)	0.65		
Severity [*n* (%)]						
≤50/100	22 (7.2)	1 (3.3)	0.43 (0.06–3.31)	0.71		
>50/100	284 (92.8)	30 (96.7)	1.00 (reference)			
Baseline GIQLI, mean (SD)	102.5 (21.2)	100.8 (25.0)	1.00 (0.99–1.02)	0.68		
Type of pain [*n* (%)]						
Episodic	180 (56.6)	19 (59.4)	0.89 (0.43–1.87)	0.76		
Non-episodic	138 (43.4)	13 (40.6)	1.00 (reference)			
Postoperative pain						
Absence	199 (61.9)	10 (31.2)	3.53 (1.62–7.71)	0.002	4.90 (2.07–11.60)	<0.001
Presence	124 (38.1)	22 (68.8)	1.00 (reference)			

*SD* standard deviation*, ASA* American Society of Anesthesiologists classification, *GIQLI* Gastrointestinal Quality of Life Index

The result of cholecystectomy was rated “excellent,” “very good” and “good” by 324 (90.0 %) patients. Baseline GIQLI score and episodic pain were associated in univariable analyses, but not in multivariable analysis (Table 4). Only postoperative absence of abdominal pain was univariably and multivariably associated with a positive result of cholecystectomy (OR 15.06, 95 % CI 4.36–52.01, *p* < 0.001).

**Table 4:** Univariable and multivariable associations of pain and patient characteristics with patient-reported result of surgery

Feature	Good result surgery	Bad result surgery	Univariable		Multivariable	
			Odds ratio (95 % CI)	*p* value	Odds ratio (95 % CI)	*p* value
Gender [*n* (%)]						
Female	249 (76.9)	29 (87.9)	0.46 (0.16–1.34)	0.16	0.76 (0.23–2.48)	0.65
Male	75 (23.1)	4 (12.1)	1.00 (reference)			
Age (years), mean (SD)	49.8 (14.1)	50.0 (14.7)	1.00 (0.98–1.03)	0.99	1.00 (0.97–1.03)	0.93
Centre [*n* (%)]						
Academic	61 (18.8)	9 (27.3)	0.62 (0.27–1.40)	0.25	0.47 (0.19–1.16)	0.10
General	263 (81.2)	24 (72.7)	1.00 (reference)			
Preoperative pain in last 2 weeks [*n* (%)]						
Absence	45 (13.9)	3 (12.1)	1.57 (0.46–5.35)	0.60	0.76 (0.20–2.95)	0.69
Presence	278 (86.1)	29 (87.9)	1.00 (reference)			
ASA [*n* (%)]						
I	154 (47.5)	13 (39.4)	1.00 (reference)			
II	170 (52.5)	20 (60.6)	0.72 (0.35–1.49)	0.37		
Duration of symptoms [*n* (%)]						
≤1 year	214 (67.7)	24 (75.0)	0.70 (0.30–1.61)	0.40		
>1 year	102 (32.3)	8 (25.0)	1.00 (reference)			
Awaking at night [*n* (%)]						
Yes	140 (44.2)	21 (65.6)	1.00 (reference)			
No	177 (55.8)	11 (34.4)	1.51 (0.70–3.24)	0.29		
Severity [*n* (%)]						
≤50/100	22 (7.1)	2 (6.7)	1.00 (reference)			
>50/100	286 (92.9)	28 (93.3)	0.93 (0.21–4.16)	1.00		
Baseline GIQLI, mean (SD)	103.0 (21.4)	95.9 (22.2)	1.02 (1.00–1.03)	0.09		
Type of pain [*n* (%)]						
Episodic	186 (41.7)	13 (40.6)	2.04 (0.98–4.23)	0.06		
Non-episodic	133 (58.3)	19 (59.4)	1.00 (reference)			
Postoperative pain						
Absence	206 (63.6)	4 (12.5)	12.2 (4.18–35.69)	<0.001	15.06 (4.36–52.01)	<0.001
Presence	118 (36.4)	28 (87.5)	1.00 (reference)			

*SD* standard deviation*, ASA* American Society of Anesthesiologists classification, *GIQLI* Gastrointestinal Quality of Life Index

### Patient-reported outcomes at 24 weeks after cholecystectomy compared to 12-week follow-up

**Fig. 2:**
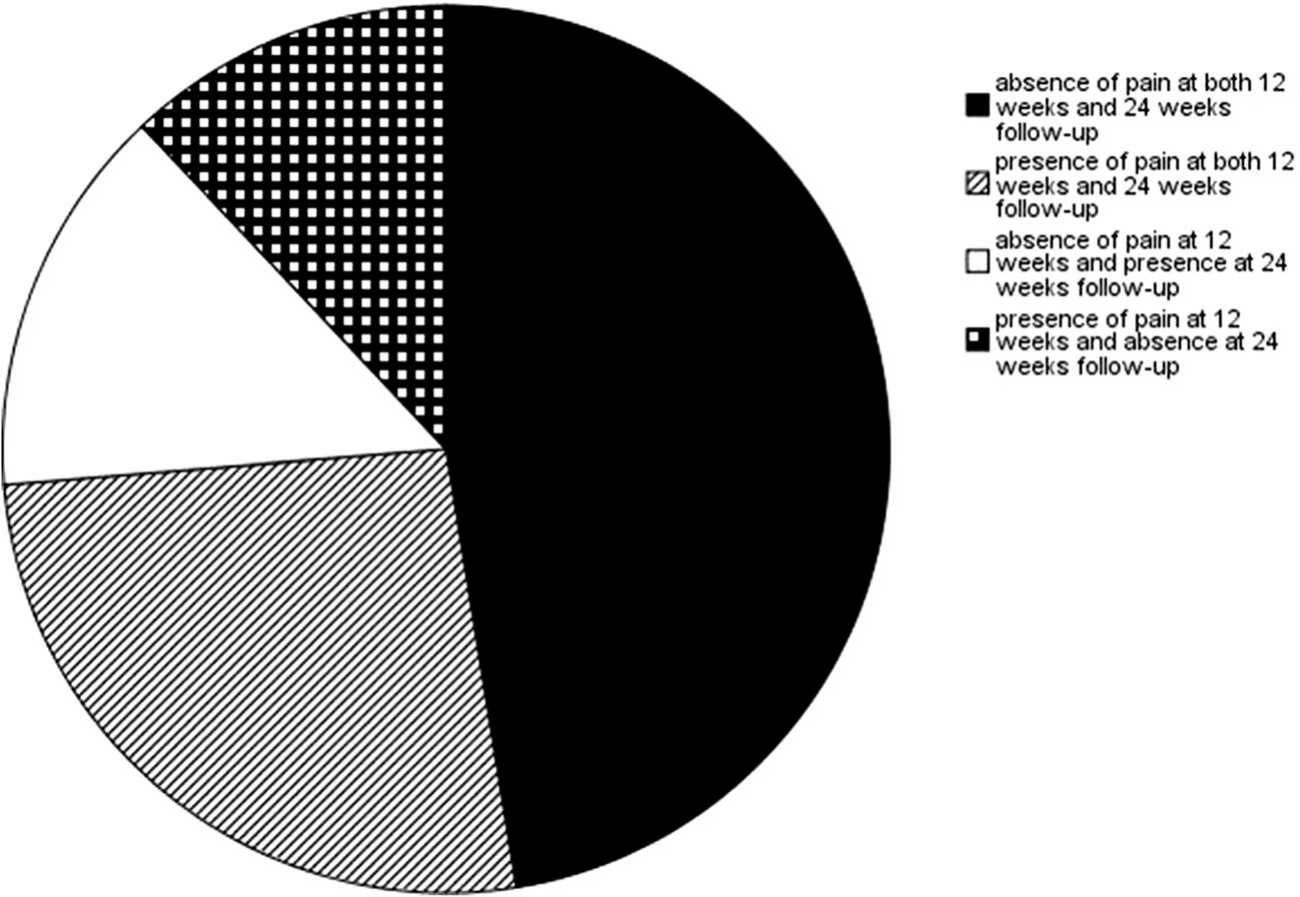
Comparison of patient-reported absence and presence of abdominal pain at 12 and 24 weeks after cholecystectomy

Three hundred and sixteen of the 360 patients who returned the follow-up questionnaire after 24 weeks had also completed the previous questionnaire at 12 weeks postoperative. There was no significant difference in the absence of abdominal pain reported at 24 and 12 weeks postoperatively (*p* = 0.51). The extent to which patients with the absence or presence of pain at 12- and 24-week follow-up were consistent is shown in Fig. 2. The patient-reported outcome on general improvement in abdominal symptoms and result of surgery was also not significantly different at 24 and 12 weeks postoperatively (*p* = 0.86 and *p* = 0.70 respectively).

Pain and patient characteristics associated with the PROMs were similar at 12 and 24 weeks, though we could not establish a significant association between preoperative episodic type of pain and the absence of pain 24 weeks after cholecystectomy.

### Associations between preoperative biliary colic and patient-reported outcomes

Of 352/360 patients, medical history of biliary colic was obtained (97.8 %). From these 352 patients, 91.7 % had a biliary colic according to the criterium: episodic pain in epigastrium or right upper abdominal quadrant for at least 30 min. 66.9 % had biliary colic with pain radiating to the back, 79.2 % had biliary colic with urge to move, and 58.3 % had biliary colic with both pain radiating to the back and urge to move. None of the criteria for biliary colic were univariably significantly associated with patient-reported absence of abdominal pain after cholecystectomy, general improvement in abdominal symptoms or result of cholecystectomy (Table 5).

**Table 5:** Univariable associations of preoperative biliary colic and postoperative patient-reported outcomes

Feature	Proportion of absence of pain (%)	Odds ratio (95 % CI)	*p* value	Improvement of symptoms (%)	Odds ratio (95 % CI)	*p* value	Positive result of surgery (%)	Odds ratio (95 % CI)	*p* value
Biliary colic									
Yes	197/329 (59.9)	1.24 (0.52–1.96)	0.62	297/326 (91.1)	1. 03 (0.23–4.06)	0.97	299/327 (91.4)	2.37 (0.75–7.50)	0.14
No	12/22 (54.4)	1.00 (reference)		20/22 (90.8)	1.00 (reference)		18/22 (81.8)	1.00 (reference)	
With pain radiating to the back									
Yes	139/240 (57.9)	0.81 (0.51–1.28)	0.36	216/239 (90.4)	0.74 (0.32–1.72)	0.49	221/240 (92.1)	1.58 (0.75–3.32)	0.23
No	70/111 (63.1)	1.00 (reference)		101/109 (92.7)	1.00 (reference)		96/109 (88.1)	1.00 (reference)	
With urge to move									
Yes	171/284 (60.2)	1.15 (0.67–1.98)	0.60	257/282 (91.1)	1.03 (0.40–2.62)	0.95	257/282 (91.1)	1.20 (0.50–2.90)	0.69
No	38/67 (56.7)	1.00 (reference)		60/66 (90.9)	1.00 (reference)		60/67 (89.6)	1.00 (reference)	
With pain radiating to the back and urge to move									
Yes	121/209 (57.9)	0.84 (0.55–1.31)	0.44	189/209 (90.4)	0.81 (0.38–1.75)	0.60	192/209 (91.9)	1.36 (0.65–2.81)	0.41
No	88/142 (62.0)	1.00 (reference)		128/139 (92.1)	1.00 (reference)		125/140 (89.3)	1.00 (reference)	

## Discussion

This prospective cohort study documents that only 59.2 % of patients are free of abdominal pain 24 weeks after cholecystectomy. Short duration of symptoms (≤1 year prior to surgery) and a higher preoperative health status predicted being pain-free after cholecystectomy. Additionally, 90 % of patients reported general improvement in abdominal symptoms and success of surgery. These PROMs were more likely to be reported by patients without abdominal pain after cholecystectomy. All PROMs were consistent in time and did not significantly differ between 12 and 24 weeks after cholecystectomy.

A recent systematic review showed patient-reported complete relief of pain after cholecystectomy in 59–100 % of patients measured between 3 and 61 weeks postoperatively [9]. The literature accords with our data and indicates that cholecystectomy is successful in 82–93 % and results in general improvement in abdominal symptoms (90 %) [15, 26–30]. Previous cohort studies showed that biliary pain (especially ≤1 per month), duration of symptoms ≤1 year prior to surgery and pain-induced awakening are associated with the absence of abdominal pain [16, 17, 19]. Presence of bloating, dyspepsia, belching, constipation or pain in the lower abdomen was associated with persistence of pain after cholecystectomy. These patients may have suffered from a concomitant functional gastrointestinal disorder [16, 18, 31–33]. A high preoperative health status indicates no or minimal symptoms besides pain. As patients describe more symptoms, GIQLI score and therefore health status decreases, which now indicates that they become less likely to be free of pain after cholecystectomy. We could not establish associations with episodic type of pain and pain-induced awakening at night. Explanations might be found in methodological differences as some studies did not include all patients before surgery, possibly introducing recall bias, and used non-validated questionnaires [16, 32]. However, PROMs are also subjective and easily influenced by patient (environmental and psychological factors) and provider characteristics (hospital- and physician-related factors), leading to variation in time [34]. To address this issue, we assessed consistency of PROMS after cholecystectomy. In line with previous studies [15, 16], we confirmed consistency of PROMs up and including 3 months after cholecystectomy. We additionally verified consistency in time of duration of symptoms ≤1 year and a higher health status with the absence of abdominal pain.

The low proportions of patient-reported postoperative pain relief suggest ineffective use of cholecystectomy. Although the Rome III criteria have been formulated to indicate symptomatic cholecystolithiasis patients and subsequent surgery, these criteria are either not sufficient to distinguish the true gallstone-related symptoms or not applied strictly by surgeons, leading to unnecessary surgeries with postoperative pain persistence. A randomized controlled trial is currently being performed to examine the latter hypothesis. Our analysis shows that the criteria for biliary colic used in common practice fail to select the gallstone patient likely to benefit from cholecystectomy. We should consider expanding current criteria to properly differentiate true gallbladder patients that benefit from surgery from non-specific patients less likely to benefit from surgery. The two factors related to positive PROMs, symptoms ≤1 year and a high preoperative health status, combined with the current biliary colic criteria, may be introduced as new selection criteria. These are the patients that should undergo cholecystectomy and are likely to be free of pain after surgery. For patients not meeting these criteria, alternative diagnoses for the abdominal symptoms should be considered and additional watchful waiting may be the suitable alternative treatment. The discrepancy between patients being free of pain (59.2 %) and reporting a successful result of surgery (90 %) however suggest that patients without the absence of pain can be satisfied with the result of surgery if other symptoms have improved. If no other diagnoses are found and patients keep experiencing symptoms, surgery may be considered to improve symptoms, although patients are less likely to be completely free of pain. This would be a shared decision of patient and physician in which the alternative goal of cholecystectomy should be clearly discussed to prevent false patients’ expectations regarding the outcome of surgery.

This study comes with strengths and limitations. The length of follow-up and repetition of measuring PROMs at longer follow-up after cholecystectomy to capture variations in responses and create a complete and reliable picture on characteristics associated with these outcomes are unique features. We also used reliable and valid questionnaires to analyze PROMs, minimizing variability in responses [34] and enhancing generalizability and comparability of the results.

The inclusion of the late responders (questionnaires completed 8–25 months postoperatively) in our analysis to maximize response rate might be viewed as a limitation. However, late responders are only a minority (12.8 %) and after exclusion of late responders data, similar results were obtained (data not shown). Another limitation is that gender and age at time of surgery and ASA classification were significantly higher among the responders than non-responders. We cannot exclude that this has affected the results, although no significant associations were seen between gender and age and the study outcomes.

In conclusion, this study establishes that the current practice based on the biliary colic as selection criteria fails to predict freedom of abdominal pain following cholecystectomy. To optimize surgical treatment for uncomplicated symptomatic cholecystolithiasis patients, criteria for cholecystectomy should be redesigned to allow use of duration of symptoms and health status as parameters. These criteria select patients likely to truly benefit from surgery and be free of pain after cholecystectomy. For other patients, cholecystectomy may be successful by improving symptoms, but they are less likely to be free of pain. PROMs are a promising tool in the evaluation of these surgical outcomes, as they show to be consistent up and including 3 months after surgery.
